# Digital volumetric assessment of CIS and tumor mass compliments conventional histopathological assessment in muscle-invasive urothelial bladder cancer

**DOI:** 10.1007/s00428-024-03875-9

**Published:** 2024-07-19

**Authors:** Fabienne Lange, Carol I. Geppert, Veronika Bahlinger, Simone Bertz, Robert Stöhr, Danijel Sikic, Helge Taubert, Sven Wach, Bernd Wullich, Arndt Hartmann, Markus Eckstein

**Affiliations:** 1 Institute of Pathology, University Hospital Erlangen, Friedrich-Alexander Universität Erlangen-Nürnberg, Krankenhausstraße. 8-10, Erlangen, 91054 Germany; 2 Comprehensive Cancer Center Erlangen-EMN (CCC ER-EMN), Erlangen, Germany; 3 Bavarian Cancer Research Center (Bayrisches Zentrum Für Krebsforschung, BZKF), Erlangen, Germany; 4 Institute of Pathology, University Hospital Tübingen, Tübingen, Germany; 5 Department of Urology and Pediatric Urology, University Hospital Erlangen, Friedrich-Alexander Universität Erlangen-Nürnberg, Erlangen, Germany

**Keywords:** Urothelial carcinoma in situ, Radical cystectomy, Prognosis, Muscle invasive bladder cancer, Urothelial carcinoma, Survival

## Abstract

Carcinoma in situ (CIS) of the bladder is a known parameter regarding the prognosis and recurrence tendency of urothelial carcinomas. Nevertheless, there is little evidence whether the amount of CIS or other precursor lesions, as well as the quantified tumor mass of muscle-invasive urothelial carcinoma, has an influence on the survival or recurrence rate of affected patients. From 80 patients with muscle invasive urothelial bladder cancer and radical cystectomy, 23 samples each were obtained as part of a whole organ mapping in a single institution study, in which the precursor lesions and tumor area were digitally measured and further correlated to pathological standard parameters, patient survival, molecular luminal and basal subtypes, and immune infiltration. Significant correlations were found between tumor mass and surface lining CIS amount for pT-stage, lymphovascular invasion, and perineural infiltration. Furthermore, an increased tumor mass as well as an increased amount of CIS combined with an increased tumor mass showed a significantly reduced survival rate in multivariable analysis (HR = 2.75; *P* = 0.019 vs. HR = 3.54; *P* = 0.002) as well as a significantly increased recurrence. No correlations could be found with molecular subtypes and immune infiltration. The exact measurement of the tumor mass with and without the CIS surface area, whether manually or, more specifically, digitally, could be incorporated into routine diagnostics and implemented as an independent predictor for patient post-surgical outcomes. It can therefore serve as an additional predictor for risk stratification and, if necessary, intensified follow-up care or therapy.

**Supplementary Information:**

The online version contains supplementary material available at 10.1007/s00428-024-03875-9.

## Introduction

Bladder cancer is among the most common cancers in the EU with an estimated incidence of 121,000 and more than 40,000 death cases per year [1]. Additionally, it is one of the overall most expensive tumor types with costs of approximately 4.9 billion € per year [1]. Men represent around three quarters of diagnosed patients, whereas women are less affected, but present more frequently with already muscle-invasive bladder cancer (MIBC) [2, 3]. Approximately 30% of newly diagnosed carcinomas are MIBC, which are usually treated with radical cystectomy (RC) and perioperative chemotherapy in curative intent [4].

MIBC is in some cases believed to develop from urothelial carcinoma in situ (CIS), which also represents the stage of intrinsic luminal and basal MIBC subtype commitment [5–8], while development from papillary tumors is rare [9]. It can also be imitated by pagetoid-ingrowing tumor cells of an invasive urothelial carcinoma, which spreads in the overlying urothelium, whereby the differential diagnosis is difficult, as CIS can also display a pagetoid pattern [10]. CIS can occur focally or diffusely and is usually not curable by endoscopic surgery [11]. Thus, CIS is a big therapeutic challenge and is usually treated with Bacillus Calmette-Guérin (BCG) instillation to prevent progression to MIBC [12, 13]. However, if BCG therapy fails, patients with persistent CIS have a poorer prognosis. Although the occurrence of CIS in NMIBC is associated with worse prognosis and increased progression risk, the role of concomitant CIS is controversially discussed in the context of MIBC: while several studies found no associations between CIS found in RC and survival [14–16], other studies demonstrated significant associations between survival and CIS in RC where the effect size decreased with increasing local tumor stage [17]. However, past studies mostly lacked standardized pathological reassessment of RC specimens and comprehensive material work-up, which is necessary to accurately assess the true prognostic impact of CIS. In the present study, we used the unique “whole bladder histological mapping” (WBHM) strategy to study the clinical impact of CIS in MIBC patients. WBHM is a unique and powerful tool, which implements comprehensive and systematic tissue sampling encompassing the entire mucosal lining and tumor mass to determine histological progression to MIBC [18, 19]. In total, we analyzed 80 consecutively collected WBHM specimens with MIBC. CIS and tumor surface areas were comprehensively measured on digitalized hematoxylin/eosin-stained slides and used to build area and volumetric CIS, tumor mass, and combined CIS/tumor mass scores which were correlated with clinico-pathological parameters and molecular subtypes and associated with long-term follow-up data.

## Patients, materials, and methods

### Patient cohort, selection, and ethical aspects

The present study was conducted based on 199 patients of the monocentric CCC-EMN cystectomy cohort who underwent RC and bilateral lymphadenectomy due to MIBC in curative intent between 2004 and 2016 without neoadjuvant therapy [18, 20, 21]. All patients were collected consecutively according to current Reporting Recommendations for Tumor Marker Prognostic Studies (REMARK) criteria. A total of 80 cystectomy specimens matched the main inclusion criterion “systematic whole organ cystectomy mapping” for study inclusion. No further exclusion or inclusion criteria were applied.

All patients gave informed consent. All experiments were performed in accordance with the declaration of Helsinki of 1975 and were approved by the local ethical review board of the FAU Erlangen-Nürnberg (approval number: #3755 and #329_16B).

### Whole bladder histological organ mapping and systematic histopathologic review

All bladder cystectomies were opened with an inverted “Y”-incision and systematically mapped according to a standard operating procedure as described previously [18]. In brief, the cystectomy is divided into 23 different areas which are embedded according to a standardized scheme (see Online Resource 1A/B). The urothelium is then submitted subtotally with relation to the muscularis and the perivesical fat with a thickness of approximately 3 mm per FFPE block. WBHM is a unique technique which allows to investigate the whole bladder mucosal lining encompassing the full spectrum of normal tumor associated urothelium, reactive changes of the epithelium, non-malignant preneoplastic lesions (hyperplasia [HYP], dysplasia [DYS]), CIS, and the whole tumor mass. A total of 1840 hematoxylin and eosin (HE)-stained sections were systematically rereviewed for urothelial abnormalities by three pathologists (FL, ME, and AH) according to the current UICC TNM staging manual (8th edition) and the WHO 2016 classification of genitourinary tumors [22]. Lesions were divided into normal appearing (i) urothelium; (ii) hyperplasia (synonym: urothelial proliferation of unknown malignant potential, UPUMP or atypical urothelial proliferation (AUP), either AUP-flat with flat architecture or AUP-tented with an implied papillary structure [23]) with an increased number of cell layers, thickened urothelium as well as none to minimal atypia; (iii) dysplasia, which is described as a flat urothelial lesion with significant architectural and cytologic abnormalities which do not fulfill the criteria of CIS; (iv) CIS, defined as a flat urothelial lesion of sometimes variable thickness with often large, pleomorphic and hyperchromatic cell nuclei; and (v) (muscle)-invasive carcinoma (Online Resource 2). Papillary tumor components were included in the carcinoma measurements, as was the intratumoral desmoplastic stroma component. Any further stromal reaction (e.g., large fibrotic areas resulting from organized tumor necrosis or post-BCG reactions) was not included in the carcinoma measurements.

### Slide scanning and digital analysis

For analysis, all 1840 HE-stained slides were scanned with a Panoramic P250 slide scanner (3DHistech) and viewed with the CaseViewer 2.0 (3DHistech). All non-invasive urothelial lesions were annotated and measured in millimeters, thus allowing to approximate the relative area occupied by the lesion, respectively. The tumor mass per HE slide was measured in mm^2^ (TM-mm^2^), exported, and used to calculate a volumetric tumor mass score for each radical cystectomy specimen. To increase comparability of the relative area occupied by CIS (CISAR) with the TM-mm^2^, both variables were Z-score transformed. The resulting normalization of the values allowed us to combine CISAR and TM into one parameter for further analysis by combining the individual *Z*-scores.

### Intrinsic subtypes and immune phenotypes

Assignments to specific intrinsic subtypes (luminal, luminal “p53”/ECM-like, basal, and double negative/DN) according to a modified MD Anderson Cancer Center (MDACC) approach and to gene expression based immune phenotypes (ImmuneTyper Cluster: Uninflamed, Inflamed: Low, Inflamed: High) were adapted from previous studies published by our group [18, 20, 21].

### Statistical analysis

Statistical analysis was performed using R (version 4.02, R Core Team [24]). Descriptive statistics were employed to characterize the distributions of continuous variables (mean, SD, SEM, quartiles, median, and range) and nominal variables (frequency, percentage). Statistical comparisons of subgroups included nonparametric Wilcoxon rank-sum test and Kruskal–Wallis test for continuous variables.

For survival analysis, Kaplan–Meier analysis was performed, and significance was tested by the log-rank test after a cut-off of low and high tumor volume was determined using a monoforest algorithm based on the prediction of disease-specific death. For each subgroup, the number of patients at risk is shown in tables below the survival plot as well as the recurrence plot. Multivariable survival analyses were conducted using Cox proportional hazards regression modeling to assess the magnitude of impact of the tumor mass combined with the CIS-surface, while adjusting for well-established prognostic clinicopathologic variables [e.g., pT-Stage, pN-Stage, lymphovascular invasion (L), age, gender, and resection margin]. *P*-values of < 0.05 were considered significant. All tests were two-sided.

## Results

### Patient characteristics

The cohort included 80 patients with MIBC, of which 63 (79%) were male and 17 (21%) female reflecting the regular sex spectrum of MIBC. Median age amounted 71 years. Detailed patient characteristics are depicted in Table 1.

**Table 1:** Clinicopathological characteristics according to different subgroups. Abbreviations: relative area occupied by carcinoma in situ (CISAR)

Characteristic	Overall (*n* = 80)	High tumor mass (*n* = 20)	Low tumor mass (*n* = 60)
Gender			
Female	17 (21.2%)	3 (15.0%)	14 (23.3%)
Male	63 (78.8%)	17 (85.0%)	46 (76.7%)
Age (years)			
Mean	69.7	72.5	68.8
Median [Min, Max]	71.0 [41.0, 91.0]	73.5 [57.0, 87.0]	70.5 [41.0, 91.0]
pT stage			
pT2	27 (33.7%)	5 (25.0%)	22 (36.7%)
pT3	39 (48.8%)	8 (40.0%)	31 (51.5%)
pT4	14 (17.5%)	7 (35.0%)	7 (11.7%)
pN stage			
pN0	53 (66.2%)	8 (40.0%)	45 (75.0%)
pN +	22 (27.4%)	10 (50.0%)	12 (20.0%)
pNX	5 (6.2%)	2 (10.0%)	3 (5.0%)
Resection margin			
R0	67 (83.8%)	15 (75.0%)	52 (86.7%)
R1	13 (16.2%)	5 (25.0%)	8 (13.3%)
Lymphovascular invasion			
L0	37 (46.2%)	7 (35.0%)	30 (50.0%)
L1	43 (53.8%)	13 (65.0%)	30 (50.0%)
Blood vessel invasion			
V0	51 (63.8%)	14 (70.0%)	37 (61.7%)
V1	29 (36.2%)	6 (30.0%)	23 (38.3%)
Perineural sheat invasion			
Pn0	48 (60.0%)	8 (40.0%)	40 (66.7%)
Pn1	32 (40.0%)	12 (60.0%)	20 (33.3%)
Subtyping			
Basal	36 (45.0%)	7 (35.0%)	29 (48.3%)
DN	5 (6.2%)	1 (5.0%)	4 (6.7%)
Luminal	31 (38.8%)	10 (50.0%)	21 (35.0%)
Luminal EMT/p53-like	8 (10.0%)	2 (10.0%)	6 (10.0%)
Tumor mass (mm^2^)			
Mean (SD)	775 (1140)	1850 (1730)	417 (496)
Median [Min, Max]	376 [0.00, 7030]	1730 [37.4, 7030]	286 [0.00, 1870]
CISAR (mm)			
Mean (SD)	60.7 (132)	191 (215)	17.4 (30.0)
Median [Min, Max]	5.78 [0.00, 841]	135 [0.00, 841]	0.00 [0.00, 124]
Immune cluster			
Inflamed: high	19 (23.8%)	4 (20.0%)	15 (25.0%)
Inflamed: low	24 (30.0%)	8 (40.0%)	16 (26.7%)
Uninflamed	37 (46.3%)	8 (40.0%)	29 (48.3%)

### Correlation of TM-mm^2^ and CISAR with clinico-pathological variables and intrinsic MIBC subtypes

Median tumor mass was 375.5 mm^2^, median CISAR 5.78 mm (Table 1). Spearman rank correlation of TM and CISAR was 0.22 (95%-CI 0.01–0.39, *P* = 0.0542) (Online Resource 3A). In addition, the Spearman rank analysis was performed to correlate the percentage of CIS on the total surface area with the tumor mass and the percentage of the tumor ulceration area to show a possible correlation. The correlations of CIS percentage with TM and the tumor ulceration area amounted 0.15 (95%-CI − 0.05–0.33, *P* = 0.19) and 0.15 (95%-CI − 0.04–0.34, *P* = 0.19), respectively (Online Resource 3B/C). To make the amount of CIS and TM-mm^2^ comparable, the standard normal random variable (*Z*-score) of both parameters was calculated and added up.

**Fig. 1:**
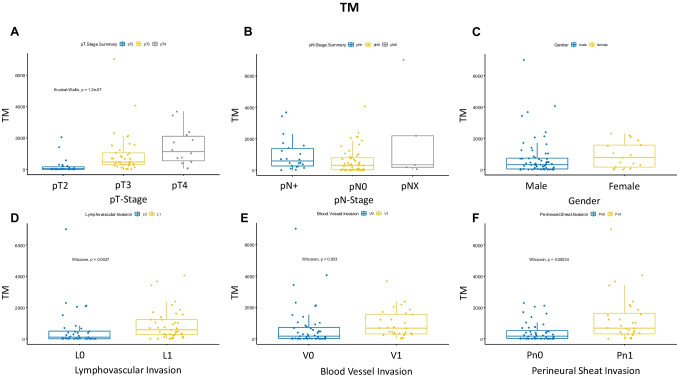
Tumor mass correlation of 80 mapping bladders with different clinical and pathological features. **A**–**F** Boxplots depicting the correlation between **A** pT stage, **B** pN stage, **C** gender, **D** lymphovascular invasion, **E** blood vessel invasioN, and **F** perineural sheath invasion. TM = tumor mass

First, we investigated which clinicopathological parameters correlate with a high TM-mm^2^. A higher TM-mm^2^ correlated significantly with a higher pT stage even though it did not correlate with a higher risk of lymph node metastasis (pN stage) (pT stage: *P* = $1.2{e}^{-07}$; pN stage: *P* = 0.09; Fig. 1A, B). Other clinicopathological parameters such as lymphovascular infiltration (L), blood vessel infiltration (V), and perineural sheath invasion (Pn) also showed a correlation with TM-mm^2^ (L: *P* = 0.0027, V: *P* = 0.003, Pn: *P* = 0.00034; Fig. 1D–F). A correlation between TM-mm^2^ and sex, immune phenotypes [18], luminal or basal subtypes, and histological subtypes could not be observed (Online Resource 4; Fig. 1C).

**Fig. 2:**
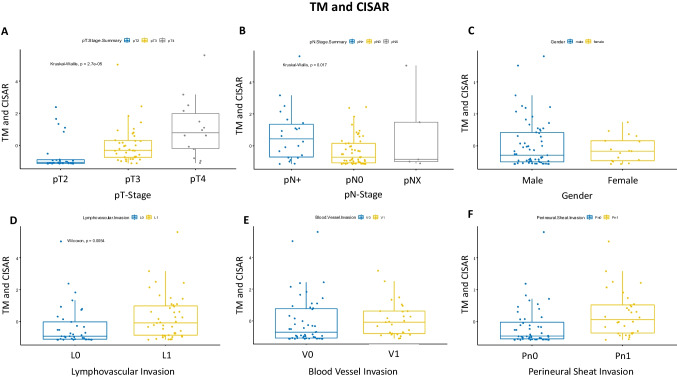
Combined *Z*-score of CIS and tumor mass association of 80 mapping bladders with different clinical and pathological features. **A**–**F** Boxplots depicting the correlation between **A** pT stage, **B** pN stage, **C** gender, **D** lymphovascular invasion, **E** blood vessel invasion, and **F** perineural sheath invasion. TM = tumor mass, CIS = carcinoma in situ

When TM and CISAR were combined (TM/CISAR), significant correlations were also found for TNM-relevant factors such as pT stage and pN stage (pT-Stage: *P* = $2.7{e}^{-05}$; pN-Stage: *P* = 0.017; Fig. 2A/B). Similar to the correlation with an increased TM-mm^2^, a correlation between TM/CISAR versus lymphovascular infiltration (L) and perineural sheath invasion (Pn) could be demonstrated (L: *P* = 0.0054, Pn: *P* = 0.00077; Fig. 2D/F). For example, 50% of patients with a low TM and CISAR showed lymphovascular invasion in contrast to 65% of patients with a high TM and CISAR (Table 1). Again, there was no correlation between sex, immune phenotypes [18], and intrinsic subtypes (Online Resource 5; Fig. 2C). In addition, a correlation between V and a high or low TM and CISAR could not be found (*P* = 0.075; Fig. 2E). In the correlation of CISAR with the clinicopathologic parameters, only a correlation between lymphonodal metastasis and CISAR could be shown; the other clinicopathologic parameters showed no significant correlation (Online Resource 6A-F).

Regarding the correlation of the different mucosal linings of the bladder and the tumor mass as well as CISAR, no significant relationship can be presented especially as far as other precursor lesions like dysplasia or lesions like hyperplasia are concerned (Online Resource 7).

### Combined TM/CISAR score and TM-mm^2^ correlates with clinical outcomes and outperforms conventional histopathological assessment

Next, we asked whether the TM-mm^2^ or the combined TM and CISAR correlated with patient outcomes. As seen in Table 1, a high TM-mm^2^ was set to be > 924.40 mm^2^ and shown in 21 patients (26%) and a low TM-mm^2^ was shown in 59 patients (74%). The cut-off for a high TM/CISAR was set at 0.6430. Both cut-offs were fitted by using monoforest algorithm based on prediction of disease-specific death. Sixty patients with a low TM/CISAR (75%) and 20 patients with a high TM/CISAR (25%) were found.

**Fig. 3:**
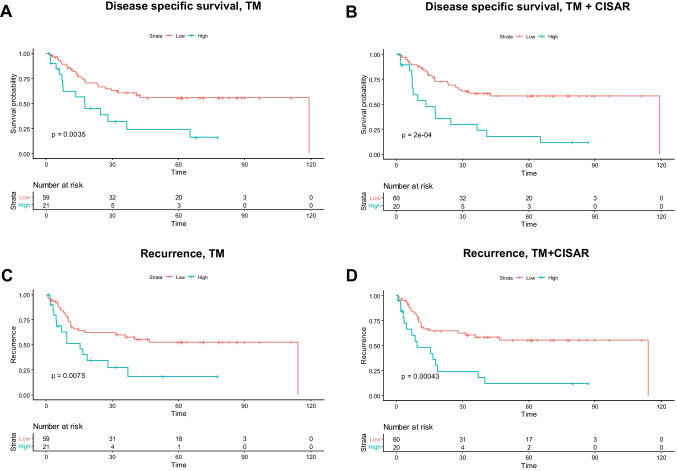
Disease specific survival and Recurrence in correlation to a high or low tumor mass or combined *Z*-score of CISAR and tumor mass. Kaplan–Meier estimates showing a significant correlation between a high combined *Z*-score of CIS and tumor mass and **A** a reduced survival time (*P* = 0.00081) as well as **C** a higher rate of recurrence (*P* = 0.00043). Same can be demonstrated between a high tumor mass and **B** the survival time (*P* = 0.0028) as well as **D** the risk of recurrence (*P* = 0.0075). TM = tumor mass, CIS = carcinoma in situ, and CISAR = area occupied by CIS

To investigate correlations between disease-specific, overall, and recurrence-free survival and a high or low TM-mm^2^ as well as the combined TM/ CISAR, univariate and multivariate analyses were performed. In the univariable analysis, the patients were divided into two groups (high TM-mm^2^ or TM/CISAR and low TM-mm^2^ or TM/CISAR) as described above. The analysis showed a significant correlation between high tumor mass (TM-mm^2^) and a reduced disease-specific (DSS), overall (OS), and recurrence free (RFS) survival time (DSS: *P* = 0.0035, OS: *P* = 0.0028; RFS: *P* = 0.0075; Fig. 3A/C). In addition, there was also a significant association of TM/CISAR and the DSS as well as the OS in the univariable Kaplan–Meier survival analysis (DSS: *P* = 0.0002; Fig. 3B, OS: *P* = 0.00081). Similar results were obtained for the univariable analysis of the RFS. Patients with a high TM-mm^2^ showed a significantly higher rate of recurrence (*P* = 0.0075; Fig. 3C), as well as patients with a high TM and CISAR (*P* = 0.00043; Fig. 3D). In the univariable survival analysis of pure CISAR, no significant correlation between CISAR and disease-specific, overall, or recurrence-free survival could be shown (DSS: *P* = 0.33; Online Resource 8A; OS: *P* = 0.44; RFS: *P* = 0.41; Online Resource 8B). The underlying cut-off was 196.26 mm, in which 71 patients had a small/low CIS area (89%) and 9 patients had a large/high CIS area (11%). To conduct the magnitude of impact of the TM-mm^2^, a multivariable survival analyses was performed (Table 2). Here, high TM-mm^2^ emerged as an independent predictor of poor overall survival (*P* = 0.019). Comparing the impact of TM-mm^2^ versus the combined TM/ CISAR score both variables emerged as independent predictors of worse overall survival depending on the amount of TM or combined TM/CISAR (hazard ratio (HR) = 2.75 TM-mm^2^ vs. HR = 3.54 TM/CISAR; Tables 2 and 3). However, the combined TM/CISAR score allows a seemingly slightly better risk stratification with higher magnitude of impact (HR = 3.54) on survival rates (*P* = 0.002; Table 2) compared to the sole analysis and evaluation of the entire TM (HR = 2.75, *P* = 0.019; Table 3). The multivariable analysis of the overall survival of the parameters is illustrated in Online Resource 9. In multivariable analysis (disease-specific survival) both TM-mm^2^ and TM/CISAR were independent predictors of decreased survival rates (HR = 3.54, *P* = 0.011 TM-mm^2^ vs. HR = 3.29, *P* = 0.012 TM/CISAR). To further compare the performance of both models, both the *C*-index and the Akaike information criterion (AIC) were assessed, where TM-mm^2^ showed a similar *C*-index and AIC (*C*-index = 0.77; AIC = 284.28) to TM/CISAR (*C*-index = 0.77; AIC = 283.70 TM/CISAR). In a model without TM-mm^2^ or TM/CISAR, the *C*-index is also slightly lower (*C*-index = 0.76; AIC = 287.75 without TM-mm^2^ or TM/CISAR).

**Table 2:** Multivariable survival analyses based on different clinical and pathological features

	Characteristic	Hazard ratio	*P*-value
TM/CISAR	Low tumor mass (*n* = 60)	-	-
	High tumor mass (*n* = 20)	3.54 (1.56–8.01)	0.002*
Age		1.01 (0.99–1.04)	0.301
Gender	Female (*n* = 17)	-	-
	Male (*n* = 63)	0.35 (0.16–0.79)	0.011*
pT-stage	pT2 (*n* = 27)	-	-
	pT3 (*n* = 39)	1.74 (0.65–4.63)	0.268
	pT4 (*n* = 14)	2.10 (0.64–6.86)	0.22
pN-stage	pN + (*n* = 22)	-	-
	pN0 (*n* = 53)	0.72 (0.31–1.68)	0.445
	pNX (*n* = 5)	2.27 (0.51–10.19)	0.283
Lymphovascular invasion	L0 (*n* = 37)	-	-
	L1 (*n* = 43)	1.41 (0.62–3.20)	0.406
Blood vessel invasion	V0 (*n* = 51)	-	-
	V1 (*n* = 29)	0.77 (0.34–1.79)	0.547
Perineural invasion	Pn0 (*n* = 48)	-	-
	Pn1 (*n* = 32)	0.97 (0.43–2.19)	0.939
Resection margin	R0 (*n* = 67)	-	-
	R1 (*n* = 13)	1.45 (0.55–3.86)	0.456
Immunetyper cluster	Inflamed: high (*n* = 19)	-	-
	Inflames: low (*n* = 24)	0.99 (0.39–2.49)	0.987
	Uninflamed (*n* = 37)	1.53 (0.62–3.79)	0.355
MDACC	Basal (*n* = 36)	-	-
	DN (*n* = 5)	1.02 (0.28–3.78)	0.973
	Luminal (*n* = 31)	0.77 (0.34–1.74)	0.529
	Luminal EMT/p53-like (*n* = 8)	3.46 (1.09–10.98)	0.035*
Events: 51; global *P*-value: 0.001079; AIC: 371.68; C-index: 0.73			

A high *Z*-score of TM and CISAR was demonstrated as an independent predictor of reduced overall survival (*P* = 0.002). *TM/CISAR* tumor mass and CISAR combined, *TM* tumor mass, *CIS* carcinoma in situ, *CISAR* area occupied by CIS, *MDACC* molecular subtyping using the classifications of the MD Anderson Cancer Center

**Table 3:** Multivariable survival analyses based on different clinical and pathological features

	Characteristic	Hazard ratio	*P*-value
TM	Low tumor mass (*n* = 59)	-	-
	High tumor mass (*n* = 21)	2.75 (1.18–6.4)	0.019*
CIS		1.00 (1.00–1.0)	0.239
Age		1.02 (0.99–1.0)	0.287
Gender	Female (*n* = 17)	-	-
	Male (*n* = 63)	0.48 (0.22–1.1)	0.069
pT-stage	pT2 (*n* = 27)	-	-
	pT3 (*n* = 39)	1.34 (0.53–3.4)	0.541
	pT4 (*n* = 14)	1.62 (0.49–5.3)	0.428
pN-stage	pN + (*n* = 22)	-	-
	pN0 (*n* = 53)	0.53 (0.22–1.3)	0.153
	pNX (*n* = 5)	3.07 (0.65–14.5)	0.157
Lymphovascular invasion	L0 (*n* = 37)	-	-
	L1 (*n* = 43)	1.44 (0.60–3.5)	0.419
Blood vessel invasion	V0 (*n* = 51)	-	-
	V1 (*n* = 29)	0.55 (0.23–1.3)	0.185
Perineural invasion	Pn0 (*n* = 48)	-	-
	Pn1 (*n* = 32)	1.04 (0.45–2.4)	0.93
Resection margin	R0 (*n* = 67)	-	-
	R1 (*n* = 13)	1.62 (0.61–4.3)	0.337
Immunetyper cluster	Inflamed: high (*n* = 19)	-	-
	Inflames: low (*n* = 24)	1.22 (0.48–3.1)	0.675
	Uninflamed (*n* = 37)	2.10 (0.81–5.4)	0.127
MDACC	Basal (*n* = 36)	-	-
	DN (*n* = 5)	1.01 (0.27–3.8)	0.99
	Luminal (*n* = 31)	0.54 (0.23–1.3)	0.177
	Luminal EMT/p53-like (*n* = 8)	2.45 (0.75–8.0)	0.14
Events: 51; global *P*-value: 0.0035714; AIC: 375.9; C-index: 0.72			

An increased TM-mm^2^ alone was significant as an independent predictor (*P* = 0.019) for overall survival. *TM* tumor mass, *CIS* carcinoma in situ, *MDACC* molecular subtyping using the classifications of the MD Anderson Cancer Center

## Discussion

Bladder cancer is a common cause of mortality and morbidity in Europe and worldwide. MIBC is in some cases believed to develop from flat precursor lesions like CIS, with similar immunohistochemical and molecular pathological characteristics [25–28]. Previous studies have controversially discussed whether tumor-associated CIS has a significant impact on clinical outcome. Some studies have demonstrated an association of concomitant CIS with recurrence as well as cancer-specific survival, while others have not demonstrated an association of MIBC with concomitant CIS [14–16]. The underlying hypothesis is often that an increased amount of CIS also increases the likelihood of aggressive behavior and thus increases, for example, the possibility of lymphovascular or blood vessel infiltration, perineural sheath infiltration as well as lymph node metastasis. However, this hypothesis could not be confirmed so far due to lack of systematic studies. Thus, the question whether the quantified amount of CIS and muscle invasive tumor plays a role in cancer specific survival or recurrence regardless of stage remained unanswered. The aim of this study was to address and answer this question by systematic quantitative analysis using digitalized whole tissue slides of whole organ mapped cystectomy specimens.

In our study, the digital analysis of 80 mapped RC revealed that an exact quantification of the tumor mass with and without the relative area occupied by concomitant CIS provides important additional information to established risk factors such as the TNM classification, intrinsic subtypes, or immune phenotypes of MIBC [20, 29]. Increased incidence of concomitant CIS in RC in combination with increased TM-mm^2^ as well as an increased TM alone demonstrates an increased risk of disease specific survival and recurrence. This may indicate that patients with increased tumor mass and CIS at enhanced risk of recurrence may benefit from increased and extended follow-up. However, the occurrence of isolated CIS is not sufficient. Since there is no significant correlation between CIS and tumor mass, the question arises whether the CIS was not present in the development of the tumor or whether, in the case of progressive tumor development, the CIS has potentially further developed into tumor. Although the first point is quite conceivable, it seems much more logical that during tumor progression, CIS may also develop into an invasive carcinoma. In addition, the amount of urothelial surface decreases with increased invasive tumor volume due to the displacement by the tumor, which may lead to a subsequent decrease of CIS surface area.

Even though analyses of TM-mm^2^ did prove to be a significant independent prognostic parameter, in principle, the combination with CISAR can have an additive effect on risk stratification if necessary. The inclusion of the surface area of the concomitant CIS seems to further enhance and enrich the already significant effect of tumor mass analysis. For example, as already touched upon, increased tumor mass entails a higher risk of lymphovascular and blood vessel infiltration. However, a significant correlation with lymph node metastasis could only be found in combination with TM and CISAR. Especially by extending the TM-mm^2^ by adding the CISAR, an improved selection of patients is possible who have an increased risk for the mentioned factors due to an associated CIS. For a pure stratification of OS or DSS, however, the mere TM-mm^2^ is also sufficient as a parameter. In bladders with a low pure tumor mass, an additive effect is possible using the combined TM/CISAR. At the background of our small cohort size, our results are exploratory, thus requiring validation and further assessment in larger cohorts of cystectomy specimens—especially to investigate the additive effect of CIS and tumor mass quantification.

In a study by Gofrit et al., a correlation between a very small tumor mass of < 1.0 cm in NMIBC and a lower recurrence rate was shown [30]. Thus, only 1.1% of patients with tumors of < 1.0 cm developed a recurrence in the following year, but 11% of patients with a larger tumor volume did. Lee et al. were able to present similar results in terms of recurrence tendency, but no association between tumor volume of NMIBC and mortality or disease progression was found [31]. Of course, results from NMIBC cannot be applied one-to-one to MIBC. However, this is a good indicator that TM-mm^2^, although relevant for prognosis estimation in the context of recurrence tendency, for example, could use supplementation. In our study, the additional evaluation of CISAR proved to be a possible supplement. Furthermore, it should be noted that the quantitative evaluation of the TM-mm^2^ and TM/CISAR adds a relevant independent impact to survival prediction beside pT stage, pN stage, sex, and age. Hence, this assessment could supplement the current practice of TN reporting and also established postoperative nomograms designed for risk stratification after radical cystectomy that mainly rely on variables such as age, sex, time from diagnosis to surgery, pathologic stage, and lymph node status [32], against which both tumor mass and TM/CISAR assessment proved to be independent predictors for decreased survival.

Interestingly, no association of TM/CISAR and TM-mm^2^ with the present molecular subtype could be shown either. Urothelial carcinomas can be divided into luminal subtypes, which have a marker profile similar to luminal urothelial cell layers, luminal “p53-/ECM”-like tumors with high gene expression of extracellular matrix remodeling related genes and basal subtypes with a squamous-like marker profile of the basal cells [33]. Strong CIS signature gene expression was demonstrated by Robertson et al. mainly in basal subtypes of MIBC, whereupon the question arises whether these developed primarily from CIS [5]. In our study, neither a relationship between TM nor between the combination of CIS and TM with the individual subtypes could be found. Rather, TM-mm^2^ and combined TM and CISAR represent a biological and prognostic factor independent from intrinsic subtypes, which can be used without further immunohistochemical studies or molecular analyses. However, a limitation for general implementation in a time of increasing workloads and shortage of pathologists is the additional effort required to assess tumors and CIS mass/volume. However, it should be noted that the 23 FFPE blocks required are of little significance compared to the time-consuming processing of a prostatectomy specimen. In addition, the quantification of the tumor mass could be automated by artificial intelligence algorithms as digitalization in pathology progresses in the next decade.

In conclusion, the current standard of pathological TN(M) staging could be supplemented by tumor mass assessment with and without quantification of CIS, which seem to be a useful and assessable histological parameter for risk stratification of MIBC patients in the future. In addition, with a significant association to recurrence, the combined TM/CISAR as well as TM-mm^2^ alone could be used as an indicator for intensified follow-up or compensatory treatment strategies after radical cystectomy.

## Limitations

Limitations of the study include its retrospective nature and limited numbers of survival events in respective analytic subgroups may have contributed to precision and power issues. Thus, upcoming studies with larger case numbers are needed to further validate the prognostic impact of digital analysis of TM-mm^2^ and TM/CISAR.

## Supplementary Information

Below is the link to the electronic supplementary material.

Supplementary file1 (PPTX 17319 KB) **Online Resource 1. Whole bladder histological organ mapping **with **(A)** standardized sampling scheme of 23 FFPE blocks and **(B) **the exemplary visualization on a cystectomy specimen with MIBC and CIS areas. MIBC = Muscle invasive bladder cancer, CIS= Carcinoma in situ. **Online Resource 2. Hematoxylin and eosin stained slides of different mucosal linings and muscle invasive urothelial bladder cancer. **Through the WBHM, it was possible to obtain an all-encompassing view of the entire mucosal lining of the cystectomies and to make an areal subdivision into **(A)** *normal urothelium*, nonmalignant preneoplastic lesions such as **(B)** *hyperplasia* and **(C)** *dysplasia*, metaplastic changes such as **(D)** *squamous metaplasia*, and malignant lesions such as **(E)** *CIS* and **(F)** *muscle invasive tumor*. WBHM= whole bladder histological mapping, CIS = Carcinoma in situ. **Online Resource 3. Correlation of CISAR and Tumor mass. (A) **Using Spearman's rank correlation, no significant correlation between CISAR and TM could be detected (*P* =0.054; R=0.22), as well as no significant correlation between the percentage of CIS on the total surface area (*P*=0.19; R=0.15; **B**) or the percentage of tumor ulceration on the total surface area (*P*=0.19; R=0.15). TM=Tumor mass, CISAR = area occupied by CIS. **Online Resource 4. Tumor mass association of 80 mapping bladders with different clinical and pathological features.** **(A-C)** Boxplots depicting the correlation or lack thereof between **(A)** *Immunetype Cluster*, **(B)** *MDACC subtyping*, **(C)** *different histological variants* and TM. TM = Tumor mass. **Online Resource 5.** **Combined Z-Score of CIS and Tumor mass correlation of 80 mapping bladders with different clinical and pathological features.** **(A-C)** Boxplots depicting the correlation or lack thereof between **(A)** *Immunetype Cluster*, **(B)** *MDACC subtyping*, **(C)** *different histological variants* and combined TM and CIS. TM = Tumor mass, CIS= Carcinoma in situ. **Online Resource 6. Correlation of the absolute values of the different urothelial mucosal lining as well as the invasive muscle invasive tumor and the combined surface of CIS and carcinoma.** Neither the CISAR nor the TM or the combined surface area of CIS and tumor showed significant correlation with the measure area occupied by normal urothelium or the nonmalignant precursor lesions such as hyperplasia and dysplasia. TM=Tumor mass, CIS = Carcinoma in situ, CISAR = area occupied by CIS. **Online Resource 7.** **Disease specific survival and Recurrence in correlation to a high or low amount of CISAR.** Kaplan-Meier estimates showing no significant correlation between a high or low amount of CISAR and **(A)** the disease specific survival time (*P*=0.33) as well as **(B)** the recurrence (*P* =0.41).

Supplementary file2 (DOCX 18 KB)

## Data Availability

The datasets used and/or analyzed during the current study are available from the corresponding author on reasonable request.
