# Supplementary material for: Digital volumetric assessment of CIS and tumor mass compliments conventional histopathological assessment in muscle-invasive urothelial bladder cancer
Source: Virchows Arch. 2024 Jul 19;486(4):769–79. doi: 10.1007/s00428-024-03875-9 (PMC12018511; doi:10.1007/s00428-024-03875-9)
Supplement: Supplementary file 1 — Supplementary file1 (PPTX 17319 KB) Online Resource 1. Whole bladder histological organ mapping with (A) standardized sampling scheme of 23 FFPE blocks and (B) the exemplary visualization on a cystectomy specimen with MIBC and CIS areas. MIBC = Muscle invasive bladder cancer, CIS= Carcinoma in situ. Online Resource 2. Hematoxylin and eosin stained slides of different mucosal linings and muscle invasive urothelial bladder cancer. Through the WBHM, it was possible to obtain an all-encompassing view of the entire mucosal lining of the cystectomies and to make an areal subdivision into (A) normal urothelium, nonmalignant preneoplastic lesions such as (B) hyperplasia and (C) dysplasia, metaplastic changes such as (D) squamous metaplasia, and malignant lesions such as (E) CIS and (F) muscle invasive tumor. WBHM= whole bladder histological mapping, CIS = Carcinoma in situ. Online Resource 3. Correlation of CISAR and Tumor mass. (A) Using Spearman's rank correlation, no significant correlation between CISAR and TM could be detected (P =0.054; R=0.22), as well as no significant correlation between the percentage of CIS on the total surface area (P=0.19; R=0.15; B) or the percentage of tumor ulceration on the total surface area (P=0.19; R=0.15). TM=Tumor mass, CISAR = area occupied by CIS. Online Resource 4. Tumor mass association of 80 mapping bladders with different clinical and pathological features. (A-C) Boxplots depicting the correlation or lack thereof between (A) Immunetype Cluster, (B) MDACC subtyping, (C) different histological variants and TM. TM = Tumor mass. Online Resource 5. Combined Z-Score of CIS and Tumor mass correlation of 80 mapping bladders with different clinical and pathological features. (A-C) Boxplots depicting the correlation or lack thereof between (A) Immunetype Cluster, (B) MDACC subtyping, (C) different histological variants and combined TM and CIS. TM = Tumor mass, CIS= Carcinoma in situ. Online Resource 6. Correlation of the absolute values of [file 428_2024_3875_MOESM1_ESM.pptx]

## Slide 1
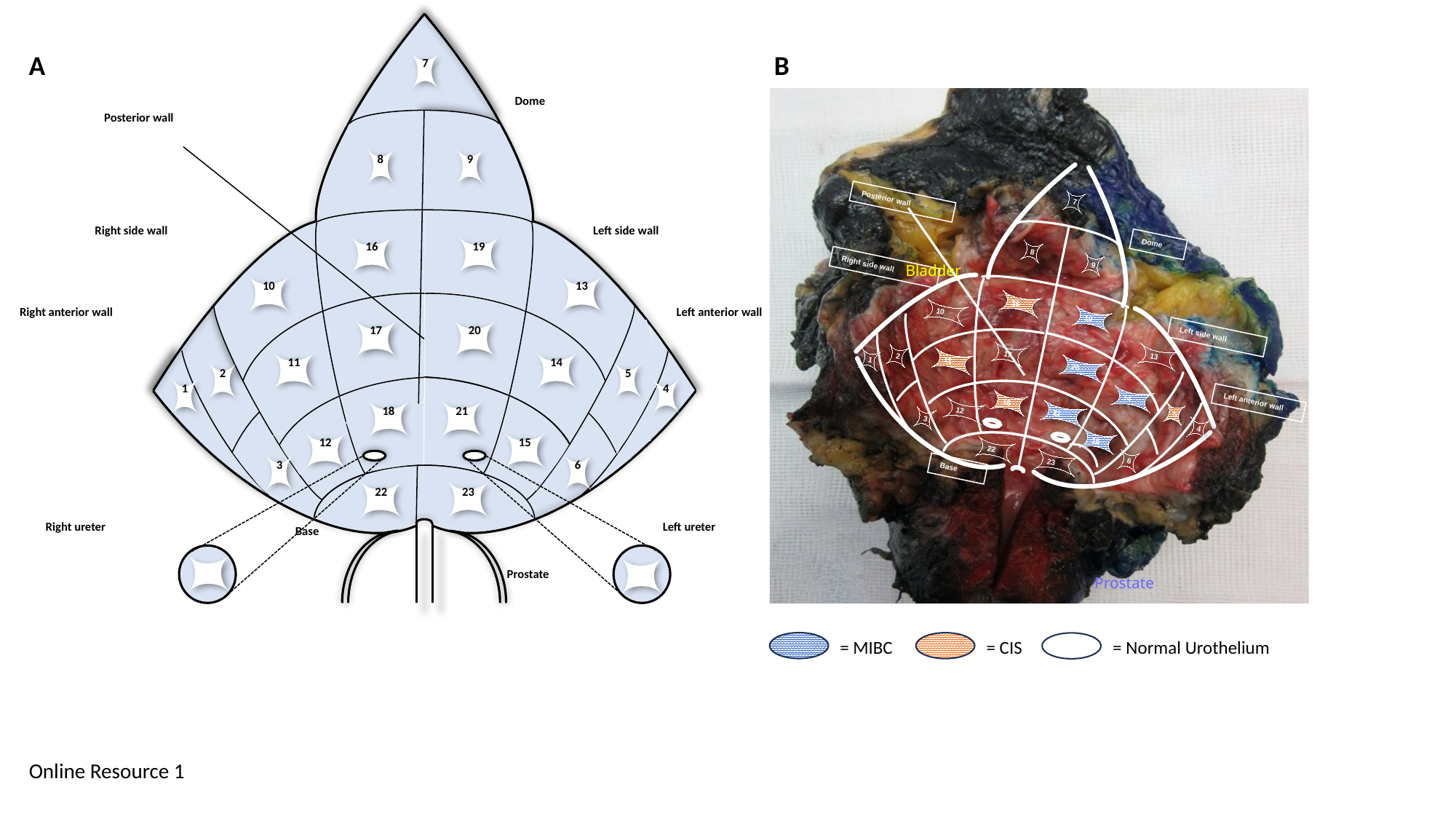

7
Dome
Posterior wall
8
9
Right side wall
Left side wall
19
16
10
13
Left anterior wall
Right anterior wall
17
20
11
14
2
5
1
4
18
21
12
15
3
6
22
23
Right ureter
Left ureter
Base
Prostate
A
B
7
Dome
Posterior wall
9
8
Left side wall
Right side wall
16
19
10
13
Left anterior wall
20
17
11
14
2
5
1
4
21
18
12
15
3
6
23
22
Base
Bladder
Prostate
= MIBC
= CIS
= Normal Urothelium
Online Resource 1

## Slide 2
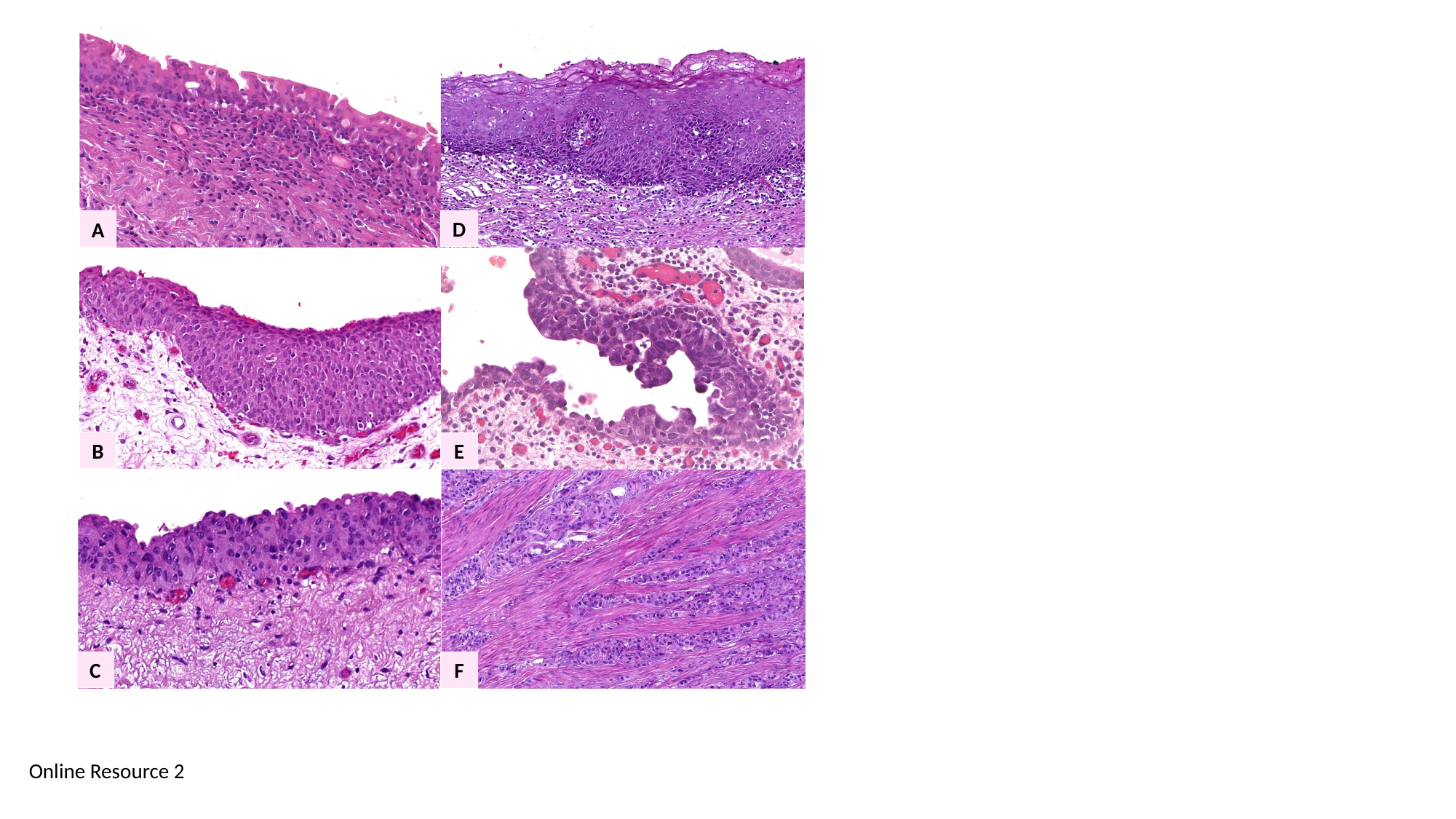

A
D
B
E
C
F
Online Resource 2

## Slide 3
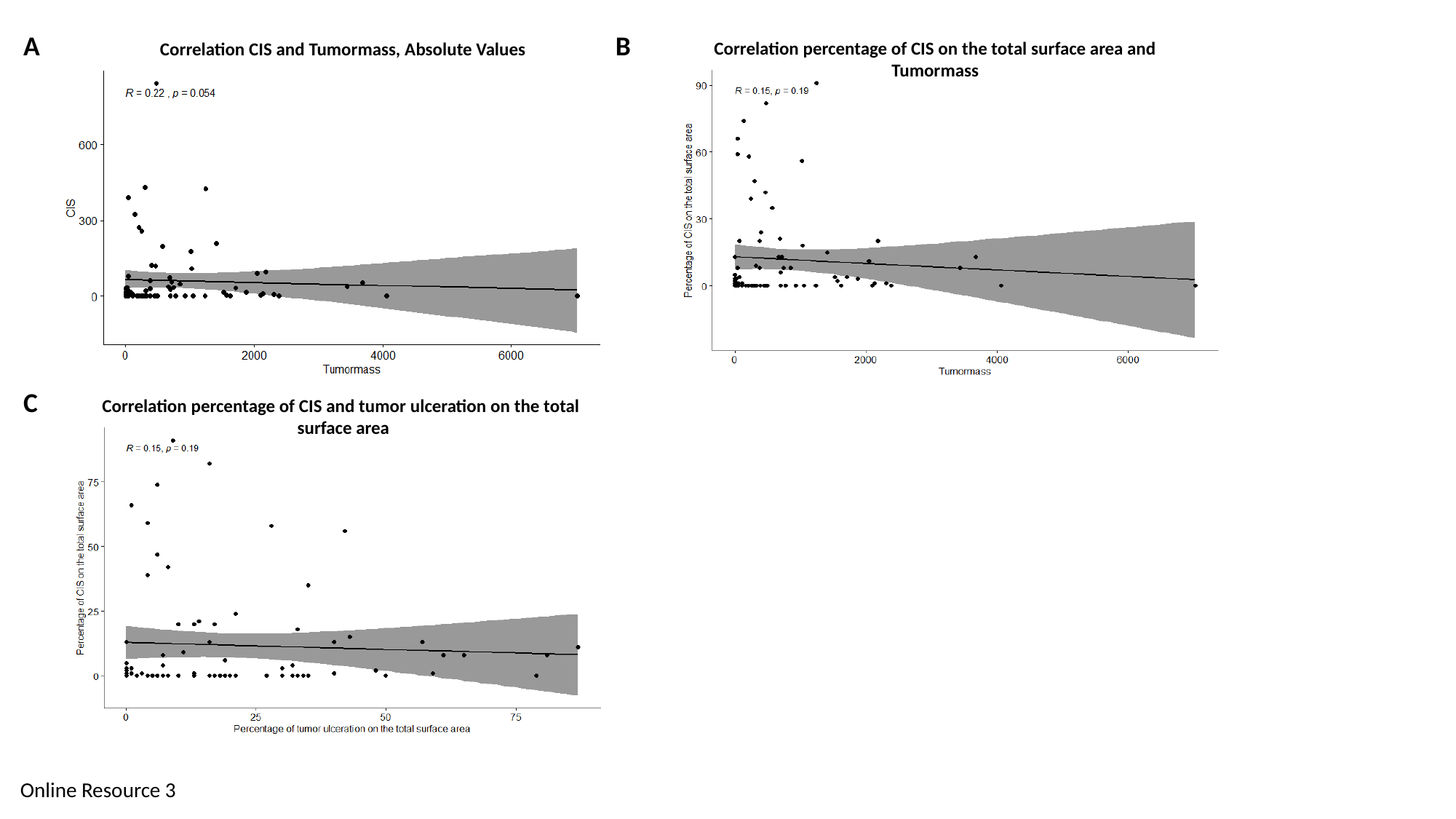

A
Correlation CIS and Tumormass, Absolute Values
B
Correlation percentage of CIS on the total surface area and Tumormass
C
Correlation percentage of CIS and tumor ulceration on the total
surface area
Online Resource 3

## Slide 4
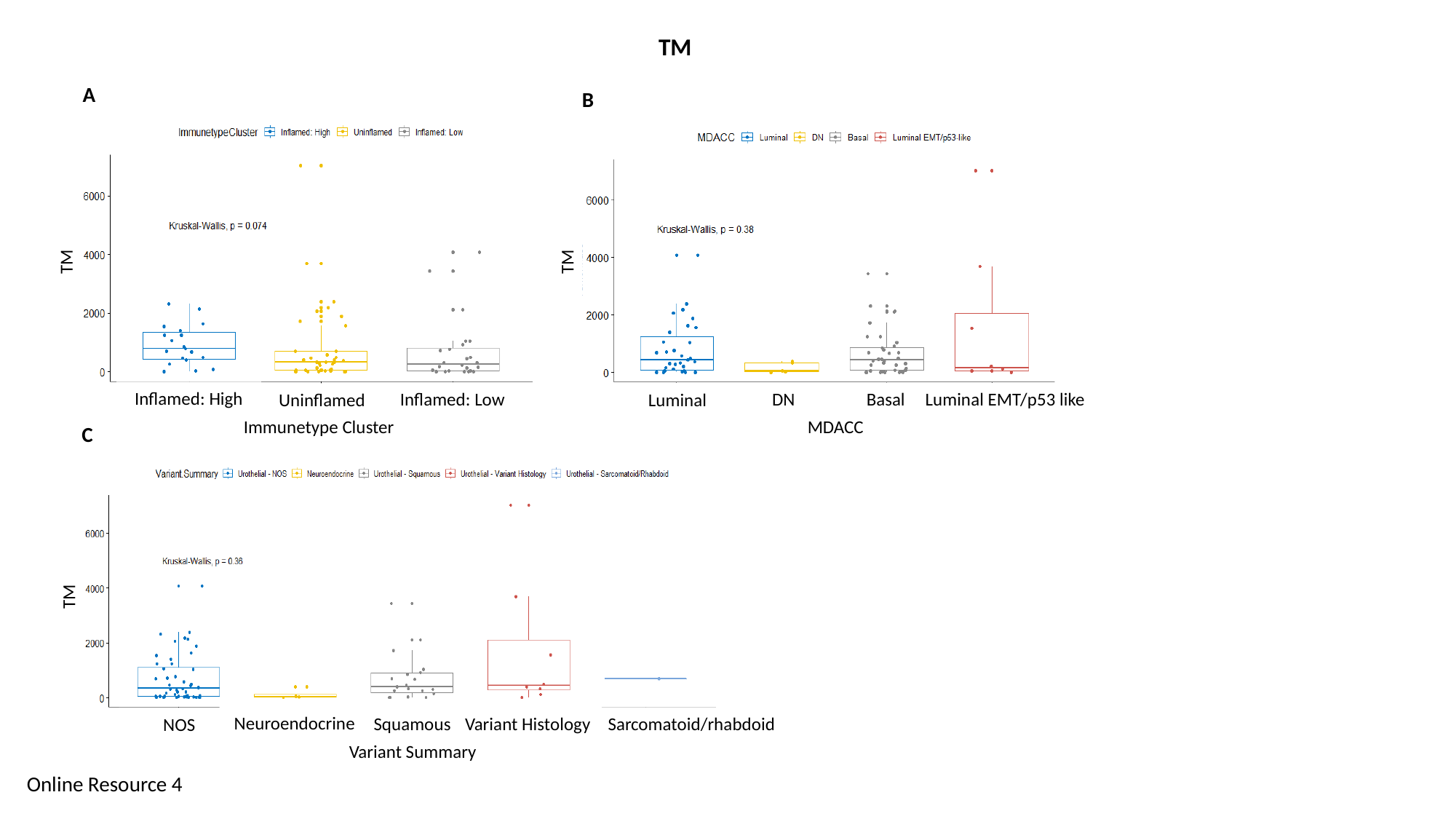

TM
A
TM
Inflamed: High
Inflamed: Low
Uninflamed
Immunetype Cluster
B
TM
DN
Basal
Luminal EMT/p53 like
Luminal
MDACC
C
TM
Neuroendocrine
Squamous
Variant Histology
Sarcomatoid/rhabdoid
NOS
Variant Summary
Online Resource 4

## Slide 5
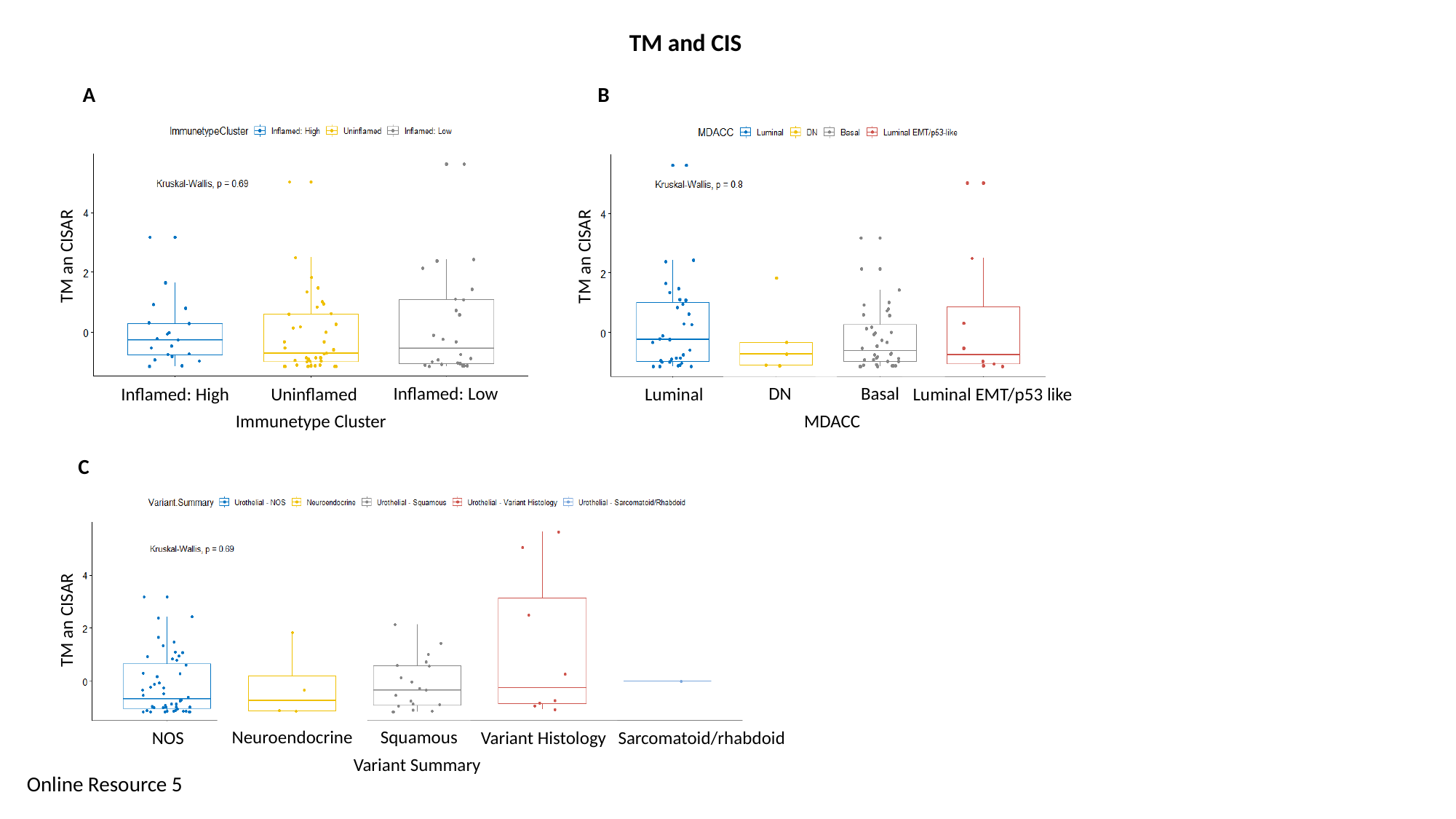

TM and CIS
A
TM an CISAR
Inflamed: Low
Inflamed: High
Uninflamed
Immunetype Cluster
B
TM an CISAR
DN
Basal
Luminal EMT/p53 like
Luminal
MDACC
C
TM an CISAR
Neuroendocrine
Squamous
Variant Histology
Sarcomatoid/rhabdoid
NOS
Variant Summary
Online Resource 5

## Slide 6
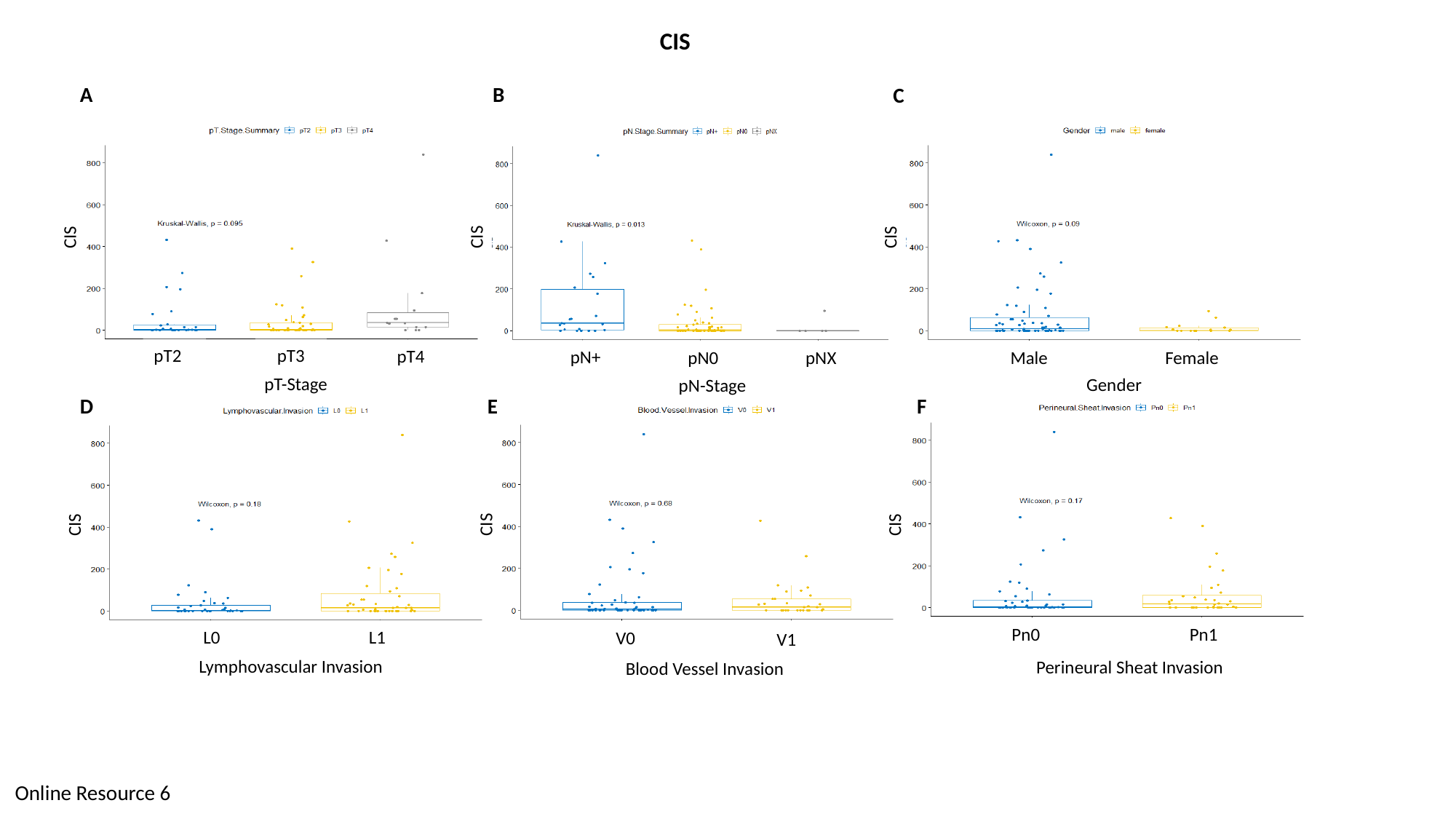

CIS
B
CIS
pN+
pN0
pNX
pN-Stage
A
pT3
pT2
pT4
pT-Stage
C
CIS
Male
Female
Gender
CIS
pN0
D
CIS
L0
L1
Lymphovascular Invasion
E
CIS
V0
V1
Blood Vessel Invasion
F
Pn0
Pn1
Perineural Sheat Invasion
CIS
Online Resource 6

## Slide 7
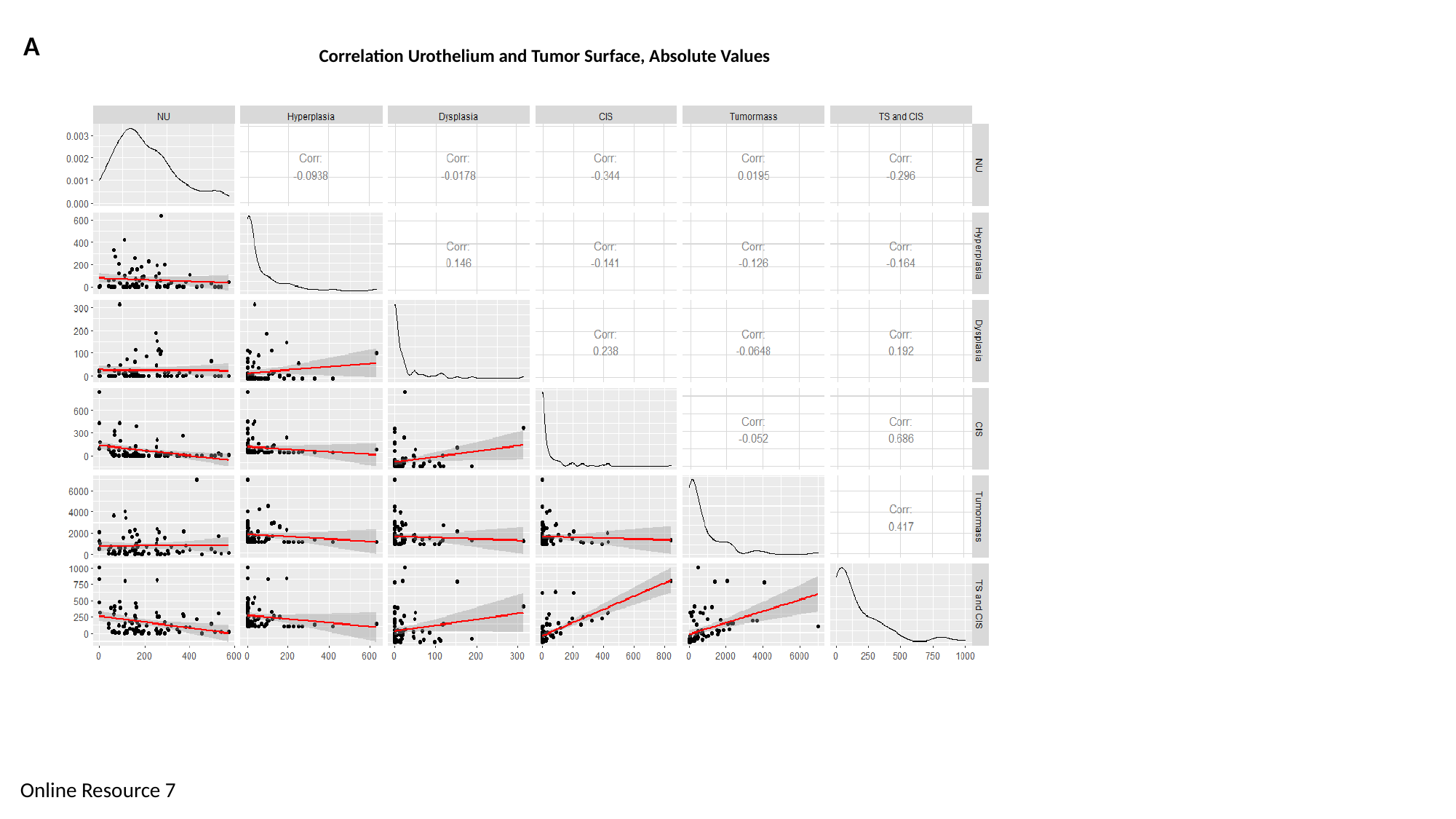

A
Correlation Urothelium and Tumor Surface, Absolute Values
Online Resource 7

## Slide 8
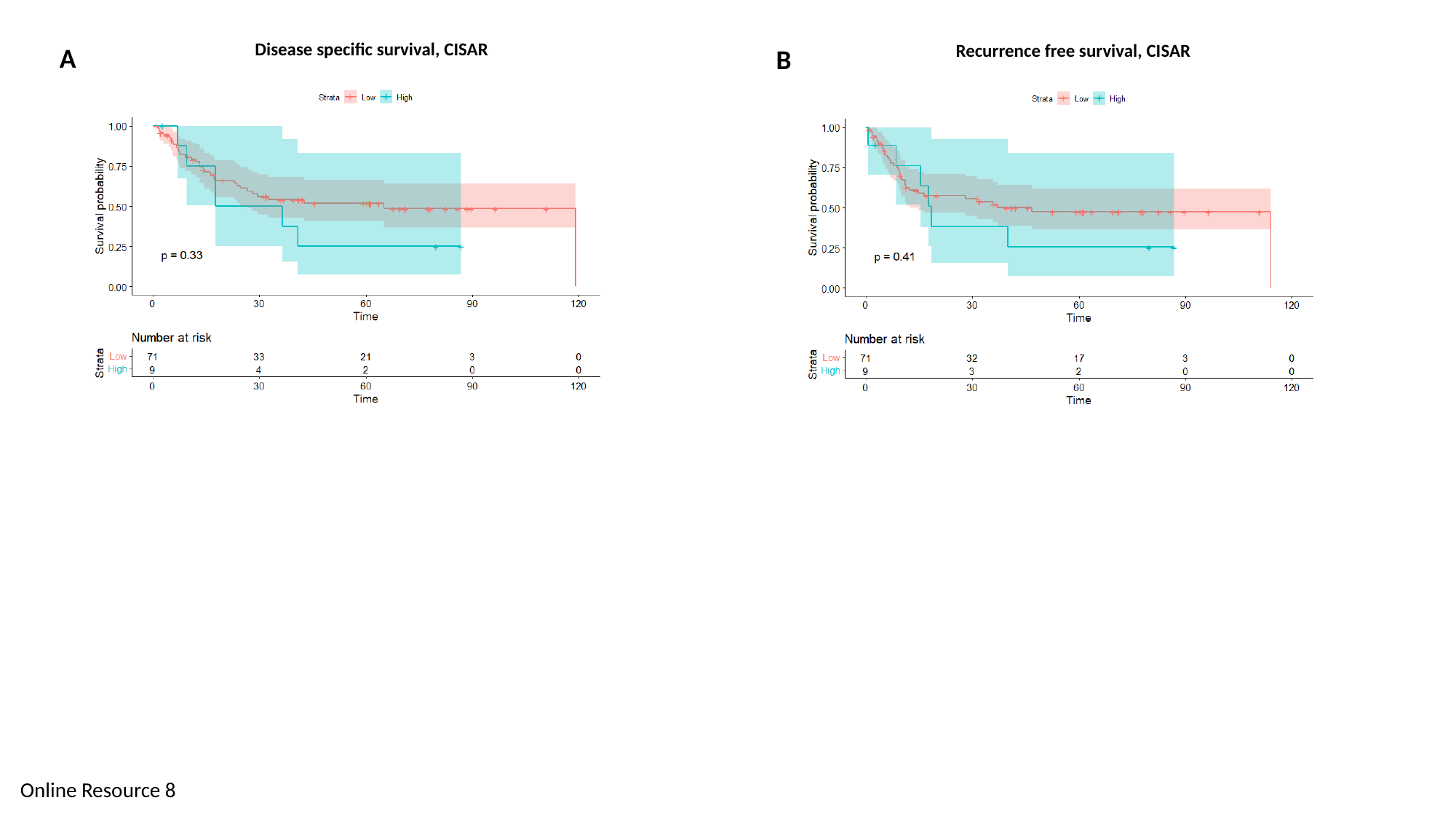

Disease specific survival, CISAR
Recurrence free survival, CISAR
A
B
Online Resource 8
