# Supplementary material for: Digital volumetric assessment of CIS and tumor mass compliments conventional histopathological assessment in muscle-invasive urothelial bladder cancer
Source: Virchows Arch. 2024 Jul 19;486(4):769–79. doi: 10.1007/s00428-024-03875-9 (PMC12018511; doi:10.1007/s00428-024-03875-9)
Supplement: Supplementary file 2 — Supplementary file2 (DOCX 18 KB) [file 428_2024_3875_MOESM2_ESM.docx]

|  | Characteristic | Hazard Ratio | P-value |  |
| --- | --- | --- | --- | --- |
| pT-Stage | pT2 (n=27) | - | - |  |
|  | pT3 (n=39) | 1.28 (0.49-3.33) | 0.616 |  |
|  | | pT4 (n=14) | 2.14 (0.66-6.94) | 0.206 |
| Age | |  | 1.02 (0.99-1.05) | 0.146 |
| Gender | | Female (n=17) | - | - |
|  | Male (n=63) | 0.44 (0.21-0.94) | 0.035* |  |
| pN-Stage | pN+ (n=22) | - | - |  |
|  | pN0 (n=53) | 0.47 (0.20-1.1) | 0.083 |  |
|  | pNX (n=5) | 1.59 (0.35-7.17) | 0.55 |  |
| Lymphovascular invasion | L0 (n=37) | - | - |  |
|  | L1 (n=43) | 1.51 (0.65-3.54) | 0.341 |  |
| Blood vessel invasion | V0 (n=51) | - | - |  |
|  | V1 (n=29) | 0.59 (0.25-1.43) | 0.32 |  |
| Perineural Invasion | Pn0 (n=48) | - | - |  |
|  | Pn1(n=32) | 1.48 (0.68-3.20) | 0.32 |  |
| Resection margin | R0 (n=67) | - | - |  |
|  | R1 (n=13) | 1.57 (0.56-4.42) | 0.39 |  |
| Immunetyper Cluster | Inflamed: High (n=19) | - | - |  |
|  | Inflames: low (n=24) | 1.10 (0.41-2.90) | 0.853 |  |
|  | Unflamed (n=37) | 1.44 (0.57-3.64) | 0.442 |  |
| MDACC | Basal (n=36) | - | - |  |
|  | DN (n=5) | 1.12 (0.30-4.12) | 0.866 |  |
|  | Luminal (n=31) | 0.67 (0.28-1.61) | 0.371 |  |
|  | Luminal EMT/p53-like (n=8) | 3.15 (1.04-9.57) | 0.043* |  |
| Events: 51; Global p-value: 0.010654; AIC: 378.34; C-Index: 0.72 | | | |  |

**Online Resource 9. Multivariable survival analyses based on different clinical and pathological features without TM or TM/CISAR for overall survival.** TM=Tumor mass. CIS = Carcinoma in situ, CISAR = area occupied by CIS.
